# Supplementary material for: The first 2 months of the SARS-CoV-2 epidemic in Yemen: Analysis of the surveillance data
Source: PLoS One. 2020 Oct 29;15(10):e0241260. doi: 10.1371/journal.pone.0241260 (PMC7595428; doi:10.1371/journal.pone.0241260)
Supplement: S1 Table — (DOCX) [file pone.0241260.s001.docx]

|  |  |  |  | Time in days from Onset of Symptoms to Admission | | | |  | Time from Admission to Death | |
| --- | --- | --- | --- | --- | --- | --- | --- | --- | --- | --- |
|  |  |  |  | cases |  |  | Deaths |  |  |  |
|  |  |  | N | Mean (Range) |  | N | Mean (Range) |  | N | Days (Range) |
| **Overall in the Country** |  |  | 268 | 5.0 (0-17) |  | 95 | 5.4 (0-17) |  | 44 | 1.1 (0-7) |
|  |  |  |  |  |  |  |  |  |  |  |
| District | Gevornorate |  |  |  |  |  |  |  |  |  |
| Al Mukalla City | Hadramaut(Al-Mukalla) |  | 61 | 5 (1-10) |  | 30 | 5 (1-12) |  | 17 | 1 (0-7) |
| Al Qahirah | Taizz |  | 22 | 5 (0-14) |  | 8 | 6 (1-14) |  | 7 | 1 (0-2) |
| Al Mudhaffar | Taizz |  | 16 | 4 (1-8) |  | 4 | 6 (2-8) |  | 3 | 2 (1-4) |
| Tarim | Hadramaut(Say'on) |  | 13 | 5 (0-12) |  | 5 | 4 (0-9) |  | 4 | 1 (1-1) |
| Tuban | Lahj |  | 15 | 4 (1-10) |  | 4 | 4 (2-6) |  | 1 | 0 (0-0) |
| Marib City | Marib |  | 9 | 4 (0-5) |  | 4 | 4 (1-7) |  | Missing | Missing |
| Ad Dhale'e | Al Dhale'e |  | 6 | 5 (3-7) |  | 2 | 5 (3-6) |  | 1 | 2 (2-2) |
| Habil Jabr | Lahj |  | 2 | 11 (7-15) |  | 2 | 11 (7-15) |  | Missing | Missing |
| Say'on | Hadramaut(Say'on) |  | 4 | 5 (2-9) |  | 1 | 6 (6-6) |  | 2 | 2 (2-2) |
| Salh | Taizz |  | 5 | 10 (1-8) |  | 2 | 14 (11-17) |  | 3 | 0 (0-0) |
| Al Had | Lahj |  | 2 | 10 (9-10) |  | 2 | 10 (9-10) |  | Missing | 0 (-) |
| Ash Shihr | Hadramaut(Al-Mukalla) |  | 6 | 8 (2-12) |  | 2 | 8 (3-12) |  | 1 | 0 (0-0) |
| Ash Shaikh Outhman | Aden |  | 4 | 3 (0-12) |  | Missing | Missing |  | 1 | 0 (0-0) |
| Jabal Habashy | Taizz |  | 2 | 7 (4-10) |  | 2 | 7 (4-10) |  | 1 | 4 (4-4) |
| Khanfir | Abyan |  | 5 | 4 (1-7) |  | 2 | 3 (2-3) |  | Missing | Missing |
| Daw'an | Hadramaut(Al-Mukalla) |  | 3 | 5 (4-6) |  | 2 | 4 (4-4) |  | 1 | 0 (0-0) |
| Radfan | Lahj |  | 2 | 8 (5-11) |  | 2 | 8 (5-11) |  | Missing | Missing |
| Others* |  |  | 91 | 4.2 (0-17) |  | 21 | 5.7 (0-13) |  | 2 | 1.8 (0-6) |

*Others, includes 43 districts that reported less than 2 confirmed cases admitted from 18 February to 5 June 2020

The table includes only confirmed SARS-CoV-2 infections admitted in the health facilities
