# Supplementary material for: The first 2 months of the SARS-CoV-2 epidemic in Yemen: Analysis of the surveillance data
Source: PLoS One. 2020 Oct 29;15(10):e0241260. doi: 10.1371/journal.pone.0241260 (PMC7595428; doi:10.1371/journal.pone.0241260)
Supplement: S2 Table — (DOCX) [file pone.0241260.s002.docx]

|  | | | | |  |
| --- | --- | --- | --- | --- | --- |
|  |  | Deaths |  | Cases |  |
|  |  | Frequency (%) |  | Frequency (%) |  |
| **Overall in the Country** |  | 111 (100) |  | 469 (100) |  |
|  |  |  |  |  |  |
| **District** | **Gevornorate** |  |  |  |  |
| Al Mukalla City | Hadramaut(Al-Mukalla) | 31 (28) |  | 73 (16) |  |
| Al Qahirah | Taizz | 9 (8) |  | 35 (7) |  |
| Al Mudhaffar | Taizz | 7 (6) |  | 31 (7) |  |
| Tarim | Hadramaut(Say'on) | 5 (5) |  | 18 (4) |  |
| Tuban | Lahj | 4 (4) |  | 15 (3) |  |
| Marib City | Marib | 4 (4) |  | 8 (2) |  |
| Ad Dhale'e | Al Dhale'e | 3 (3) |  | 10 (2) |  |
| Habil Jabr | Lahj | 3 (3) |  | 3 (1) |  |
| Say'on | Hadramaut(Say'on) | 3 (3) |  | 6 (1) |  |
| Salh | Taizz | 3 (3) |  | 8 (2) |  |
| Al Had | Lahj | 2 (2) |  | 2 (0) |  |
| Ash Shihr | Hadramaut(Al-Mukalla) | 2 (2) |  | 6 (1) |  |
| Ash Shaikh Outhman | Aden | 2 (2) |  | 14 (3) |  |
| Jabal Habashy | Taizz | 2 (2) |  | 4 (1) |  |
| Khanfir | Abyan | 2 (2) |  | 9 (2) |  |
| Daw'an | Hadramaut(Al-Mukalla) | 2 (2) |  | 5 (1) |  |
| Radfan | Lahj | 2 (2) |  | 2 (0) |  |
| Others* |  | 25 (25) |  | 99 (21) |  |
