# Supplementary material for: The first 2 months of the SARS-CoV-2 epidemic in Yemen: Analysis of the surveillance data
Source: PLoS One. 2020 Oct 29;15(10):e0241260. doi: 10.1371/journal.pone.0241260 (PMC7595428; doi:10.1371/journal.pone.0241260)
Supplement: Attachment — Submitted filename: Response to Reviewers.docx [file pone.0241260.s004.docx]

Response to the comments made by Reviewer 1

“While there were not overt methodological limitations, there was limited data available and biased sample collection beyond the control of study staff, however interpretation of the limited data available has now been re-framed to reflect weaknesses in data collection and is presented in the appropriate context.”

Response: *Many thanks for your time and the valuable comments that improved our manuscript.*

Response to the comments made by Reviewer 2

“- Abstract conclusion: Authors are invited to present their results within the sampling frame of the Southern and Eastern part of Yemen.”

Response: *Thank you for the comments. We have specified in the abstract conclusion the sampling frame of the southern and eastern governorates.*

“Line 78 : Authors should acknowledge the potential sampling and under-reporting impact on the results to nuance the picture of the two first months”

Response: *Many thanks for this observation. We totally agree that the potential sampling and under-reporting impact on the results should be acknowledged. It is extensively mentioned in the discussions and limitations of our study. Now we have included also in the abstract as suggested*

“Line 219: Authors are invited to explain and add a reference to table S1”

Response: *Thank you very much for this suggestion. The reference to table S1 is already in line 251. We have included the explanation of the table as suggested and additional reference in the results sections. In addition, we have corrected a minor error in the totals on the same S1 table that does not affected the results and conclusions in the manuscript.*

“Line 282-287: Authors should state that the presented descriptive statistics are representative of the southern and Eastern part.”

Response: *Many thanks again for this observation. We have included the statement as suggested*

“Line 359-360: The reference to high altitude and low temperature could lead to think the two variables could have a possible effect on COVID-19 transmission and profile. This should be referenced or edited accordingly.”

Response: *Thank you for this excellent observation. We have included two references of effects of temperature and altitude on COVID-19 transmission profile.*
